# Development and validation of a set of six adaptable prognosis prediction (SAP) models based on time-series real-world big data analysis for patients with cancer receiving chemotherapy: A multicenter case crossover study

**DOI:** 10.1371/journal.pone.0183291

**Published:** 2017-08-24

**Authors:** Yu Uneno, Kei Taneishi, Masashi Kanai, Kazuya Okamoto, Yosuke Yamamoto, Akira Yoshioka, Shuji Hiramoto, Akira Nozaki, Yoshitaka Nishikawa, Daisuke Yamaguchi, Teruko Tomono, Masahiko Nakatsui, Mika Baba, Tatsuya Morita, Shigemi Matsumoto, Tomohiro Kuroda, Yasushi Okuno, Manabu Muto

**Affiliations:** 1 Department of Clinical Oncology, Kyoto University Hospital, Kyoto city, Japan; 2 RIKEN Advanced Institute for Computational Science, Kobe city, Japan; 3 Division of Information Technology and Administration Planning, Kyoto University Hospital, Kyoto city, Japan; 4 Department of Healthcare Epidemiology, School of Public Health in the Graduate School of Medicine, Kyoto University, Kyoto city, Japan; 5 Institute for Advancement of Clinical and Translational Science, Kyoto University Hospital, Kyoto city, Japan; 6 Department of Palliative Care, Mitsubishi Kyoto Hospital, Kyoto city, Japan; 7 Department of Clinical Oncology, Mitsubishi Kyoto Hospital, Kyoto city, Japan; 8 Department of Medical Oncology, Kyoto Min-iren Chuo Hospital, Kyoto city, Japan; 9 Department of Gastrointestinal Oncology, National Cancer Center Hospital East, Kashiwa city, Japan; 10 Department of Gastroenterology and Hepatology, Graduate School of Medicine, Kyoto University, Kyoto city, Japan; 11 Department of Clinical System Onco-Informatics, Graduate School of Medicine, Kyoto University, Kyoto city, Japan; 12 Department of Palliative Medicine, Suita Tokushukai Hospital, Suita city, Japan; 13 Palliative and Supportive Care Division, Seirei Mikatahara General Hospital, Hamamatsu city, Japan; China Medical University, TAIWAN

## Abstract

**Background:**

We aimed to develop an adaptable prognosis prediction model that could be applied at any time point during the treatment course for patients with cancer receiving chemotherapy, by applying time-series real-world big data.

**Methods:**

Between April 2004 and September 2014, 4,997 patients with cancer who had received systemic chemotherapy were registered in a prospective cohort database at the Kyoto University Hospital. Of these, 2,693 patients with a death record were eligible for inclusion and divided into training (n = 1,341) and test (n = 1,352) cohorts. In total, 3,471,521 laboratory data at 115,738 time points, representing 40 laboratory items [e.g., white blood cell counts and albumin (Alb) levels] that were monitored for 1 year before the death event were applied for constructing prognosis prediction models. All possible prediction models comprising three different items from 40 laboratory items (_40_C_3_ = 9,880) were generated in the training cohort, and the model selection was performed in the test cohort. The fitness of the selected models was externally validated in the validation cohort from three independent settings.

**Results:**

A prognosis prediction model utilizing Alb, lactate dehydrogenase, and neutrophils was selected based on a strong ability to predict death events within 1–6 months and a set of six prediction models corresponding to 1,2, 3, 4, 5, and 6 months was developed. The area under the curve (AUC) ranged from 0.852 for the 1 month model to 0.713 for the 6 month model. External validation supported the performance of these models.

**Conclusion:**

By applying time-series real-world big data, we successfully developed a set of six adaptable prognosis prediction models for patients with cancer receiving chemotherapy.

## Introduction

Prognosis prediction is one of the most clinically relevant issues for both physicians and patients with cancer. Accurate prognosis prediction can help physicians select the optimal treatment (e.g., discontinuation of aggressive interventions at the end-of-life). It is well-known that patients’ understanding of their prognosis is poor, [1, 2] and overestimation of their life expectancy could positively affect the preference for more aggressive treatments. [3,4]

Many attempts have been made to address this issue, and several prognosis prediction models have been proposed: the palliative prognostic index (PPI), palliative prognostic (PaP) score, delirium-palliative prognostic (D-PaP) score, and prognosis in palliative care study (PiPS) models. [5–8] However, these models were developed using the variables from a single time point (e.g., admission date or baseline assessment date), which limits the use of these models under baseline conditions. Because patient’s condition during the treatment course can change from the baseline, the development of an adaptable prognosis prediction model that could be applied at any time point after the initiation of chemotherapy, is eagerly warranted in daily clinical practice.

To develop adaptable prognosis prediction models using laboratory variables, we used every serial laboratory variable monitored within 1 year before a death event as time-inclusive data.

## Patients and methods

### Study population and data collection

**Fig 1:**
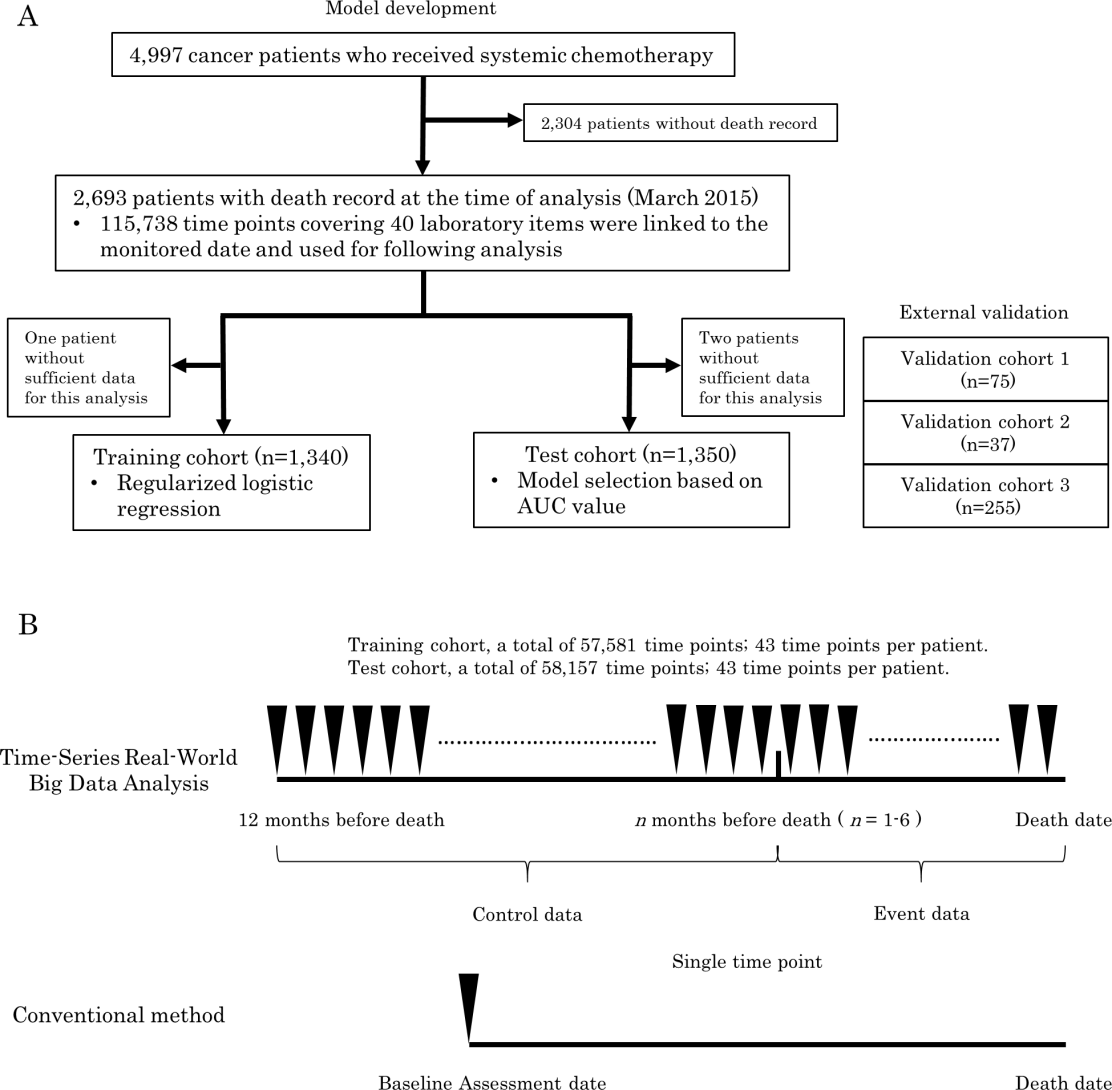
(A) Flow of the study. (B) Comparison of time-series real-world big data analysis with conventional methods. Upper: Time-series real-world big data analysis included every time point monitored within 1 year before the death event in the analysis as an explanatory variable. Each laboratory variable was used as time-inculsive data, classified event data and control data bounded by *n* months before the date death. Lower: The conventional method involved single time point (such as admission date or baseline assessment date) as an explanatory variable. AUC, area under the curve; ROC, receiver operating characteristic curve; Black arrow, explanatory variable.

Between April 2004 and September 2014, a total of 4,997 patients with cancer who had received systemic chemotherapy were registered in the prospective cohort database (CyberOncology^®^, Cyber Laboratory Inc., Tokyo, Japan) at the Kyoto University Hospital. [9] (Fig 1A) Among them, 2,693 patients with death records at the time of this analysis (March 2015) were considered eligible for this study (Table 1). These patients were randomly divided into training (*n* = 1,341) and test cohorts (*n* = 1,352). One patient from the training cohort and two from the test cohort were excluded because of insufficient data for this analysis. (Fig 1A) In total, 7,606,544 laboratory data at 159,316 time points covering 1,147 items [e.g., white blood cell counts and albumin (Alb) levels] that had been monitored within 1 year before death events were retrieved from CyberOncology^®^ and electronic medical records. To improve the feasibility of the models in daily clinical practice, we considered that it was preferable to be comprised of routinely monitored laboratory items. Therefore, we excluded items monitored at less than 50% of all time points (e.g., tumor markers) and urine-based data. Finally, 3,471,521 laboratory data at 115,738 time points covering 40 laboratory items were utilized for constructing prognosis prediction models. (Fig 1A) For external validation, we enrolled 75 patients corresponding to 1,581 laboratory data at 527 time points from the Mitsubishi Kyoto Hospital between February 2014 and August 2014, 37 patients corresponding to 582 laboratory data at 194 time points from the Kyoto Min-iren Chuo Hospital between April 2014 and May 2015 and 255 patients corresponding to 765 laboratory data at 255 time points from the Japan prognostic assessment tools validation (J-ProVal) study between September 2012 and April 2014. [10] (S1 Table) The J-ProVal study was a multicenter prospective cohort study involving a total of 58 palliative care facilities in Japan. We included the patients who were receiving chemotherapy from the J-ProVal study cohort. This study was approved by the institutional review boards of the Kyoto University Hospital (E2200) and other participating hospitals.

**Table 1:** Patient characteristics.

Factor	Number
Sex	
Female	987 (36.7%)
Male	1,706 (63.3%)
Age (years)	
≥65	1,531 (56.9%)
<65	1,162 (43.1%)
Primary site	
Lung	862 (35.8%)
Pancreas	374 (15.5%)
Colon/rectum	291 (12.1%)
Stomach	245 (10.2%)
Breast	159 (6.6%)
Esophagus	138 (4.6%)
Bile duct	122 (5.1%)
Lymph node	120 (5.1%)
Liver	97 (4.0%)
Others	284 (9.5%)
MST (month, range)	16.2 (0.2–142.4)
MST 95% CI	15.5–16.7
Prior treatment	
Surgery	964 (35.8%)
Radiation	874 (32.5%)

MST, median survival time; 95% CI, 95% confidence interval

### Statistical analysis

#### Development of adaptable prognosis prediction models in the training cohort

First, we linked 1,730,535 laboratory variables to 57,581 time points and used these time-inclusive data. We aimed to develop a set of adaptable prognosis prediction models that could predict death events within *n* months (*n* = 1–6). Therefore, when constructing such model, laboratory variables monitored within *n* months before the death event were classified as event data, whereas other data monitored between *n* + 1 and 12 months before the event were classified as control data. [11] (Fig 1B) Because the positive predictive value (PPV) and negative predictive value (NPV) are affected by imbalances in the sample size between the control and event data, they were adjusted using the oversampling method to make PPV and NPV interpretable. [12, 13]

We considered that three explanatory variables were feasible for the developed model to be generalizable and easy-to-use in daily clinical practice. Therefore, all possible combinations of three different items (e.g., C-reactive protein, calcium, and total protein) from 40 laboratory items (_40_C_3_ = 9,880) were generated; the same number of prognosis prediction models comprising three different items were tested using regularized logistic regression analysis, and the coefficients of explanatory variables were calculated. [12] Because our current prognostic models handled the discrimination of binary survival outcomes within *n* months (*n* = 1–6), we considered that logistic regression was more feasible than the Cox regression model.

#### Model selection in the test cohort

The area under the curve (AUC) and cutoff value were estimated via receiver operating characteristic (ROC) curve analysis in the test cohort comprising 1,740,986 laboratory data at 58,157 time points. Model selection was performed on the basis of the AUC values derived from prognosis models predicting death events within *n* months (*n* = 1–6).

#### External validation of selected models

External validation was performed using 2,163 laboratory data at 721 time points from the two independent community hospitals and 765 laboratory data at 255 time points from J-ProVal study cohort. Because the participants in the J-ProVal study who survived for more than 180 days were censored, the validation of the six month model was skipped for the J-ProVal study cohort.

All statistical analyses were performed using GNU R software (version 3.2.0; R Project for Statistical Computing, Vienna, Austria) and Python (version 3.5.1; Python Software Foundation).

## Results

### Development of prognosis prediction models

**Fig 2:**
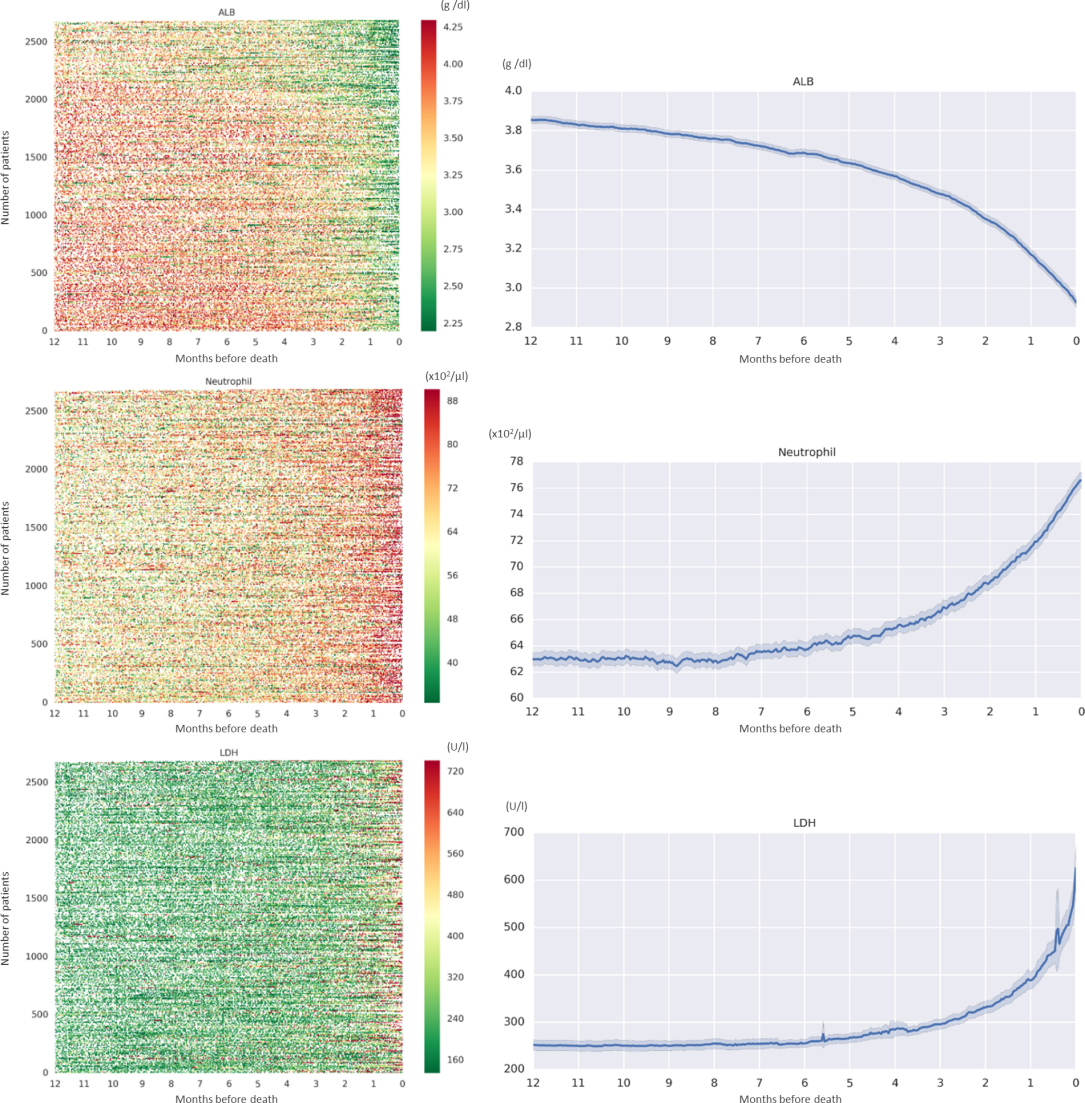
Left: Time-series heat map transition of albumin (Alb), neutrophil (Neu), and lactate dehydrogenase (LDH) levels. Right: Mean Alb, Neu, and LDH levels with 95% confidence intervals.

A total of 59,280 prediction models comprising three laboratory items for death events within *n* months (*n* = 1–6) were generated using the training cohort (9,880 models per each prediction month). The performance of these models was evaluated on basis of AUC using the test cohort. Among the top 10 prediction models based on AUC values, the combination of Alb, lactate dehydrogenase (LDH), and neutrophil (Neu) levels yielded the best and second best prediction performance at four (2, 3, 4, and 5 months) and two (1 and 6 months) of the six tested time points, respectively, (S2 and S3 Tables) and was therefore selected as our prognosis prediction model. A time-series heat map of the three items (Alb, LDH, and Neu) revealed gradual increases in LDH and Neu levels and a decrease in Alb as patients approached the death event. (Fig 2) AUC, sensitivity, specificity, PPV, NPV, and accuracy of prediction model corresponding to each prediction period (1, 2, 3, 4, 5, and 6 months) are summarized in Table 2, and the regression equations are summarized in Table 3.

**Table 2:** Performance of albumin, lactate dehydrogenase, and neutrophil models for prediction of death events within 1–6 months.

Prediction Period	Area under the curve	Number of applied data	Patients	Sensitiviy	Specificity	PPV	NPV	Accuracy
Death event within 1 month	0.852	76,642	1,350	0.786	0.78	0.781	0.785	0.783
Death event within 2 months	0.808	68,822	1,350	0.749	0.74	0.742	0.746	0.744
Death event within 3 months	0.774	60,686	1,350	0.687	0.741	0.726	0.703	0.714
Death event within 4 months	0.754	53,008	1,350	0.649	0.743	0.716	0.679	0.696
Death event within 5 months	0.733	45,132	1,350	0.627	0.732	0.701	0.662	0.68
Death event within 6 months	0.713	45,554	1,350	0.589	0.732	0.687	0.64	0.66

PPV, positive predictive value; NPV, negative predictive value

**Table 3:** Regression equation corresponding to each prediction period. $p=1/\left(1+\exp(-c_{\mathrm{Alb}}\times_{\mathrm{Alb}}-c_{\mathrm{LDH}}\times_{\mathrm{LDH}}-c_{\mathrm{Neutrophil}}\times_{\mathrm{Neutrophil}}-\mathrm{const}.)\right)$

	Coefficients	Albumin	Lactate dehydrogenase	Neutrophil	Const.	Cutoff
Months						
1		-0.701	0.002	0.023	-0.051	0.496
2		-0.573	0.002	0.02	-0.042	0.488
3		-0.482	0.001	0.017	-0.039	0.501
4		-0.407	0.001	0.015	-0.031	0.497
5		-0.347	0.001	0.013	-0.033	0.507
6		-0.334	0.001	0.012	-0.031	0.503

Alb, albumin; LDH, lactate dehydrogenase.

### Predictive performance among different tumor types

**Fig 3:**
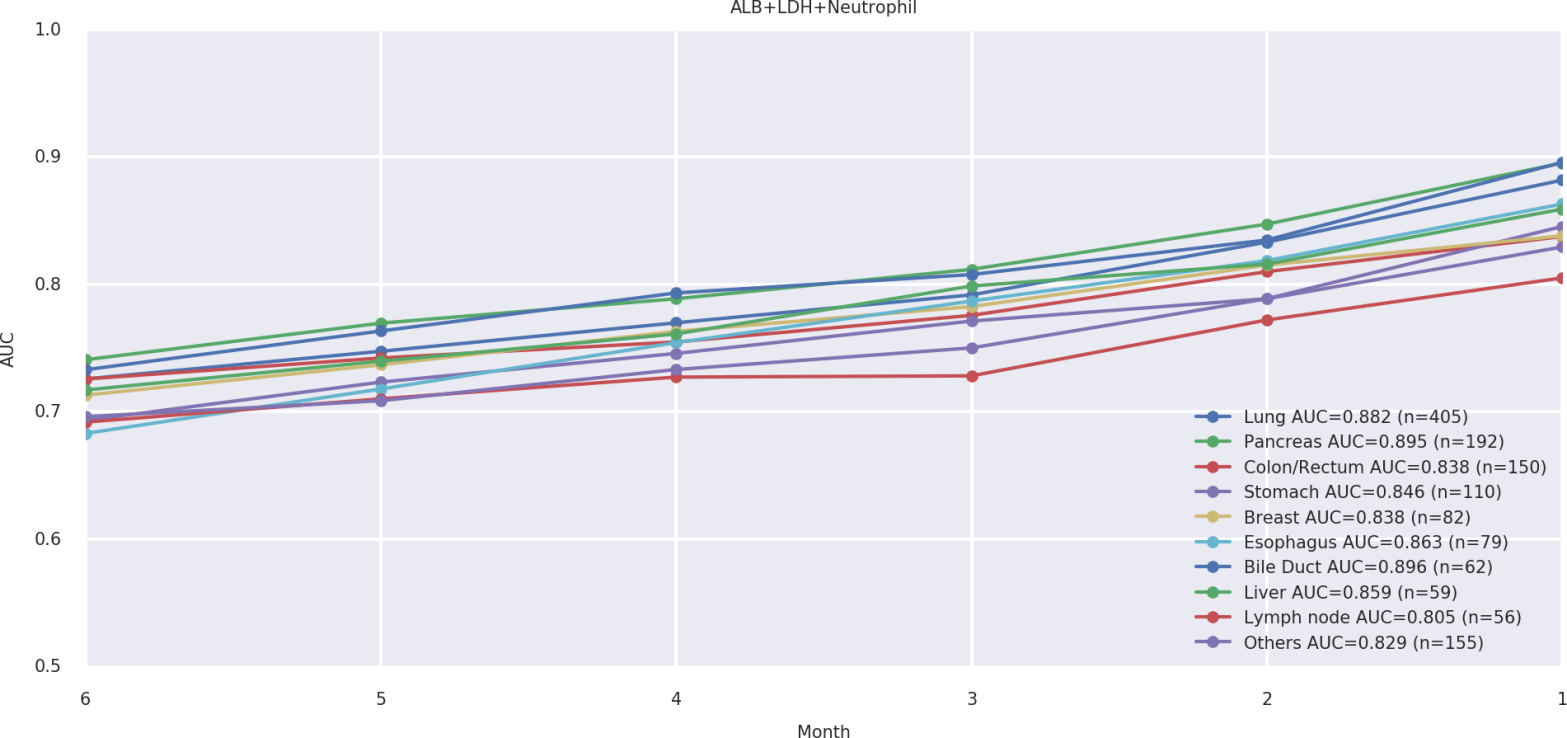
Area under the receiver operating characteristic curve values for the prediction of death events within 1–6 months among 10 different tumor types.

Next, we tested the fitness of these prediction models across 10 different tumor types. With all tumor types, our models yielded a high level of predictive performance, with AUC values consistently > 0.750 for 1 and 2 months before death events (Fig 3).

### External validation of new prognosis prediction models

**Fig 4:**
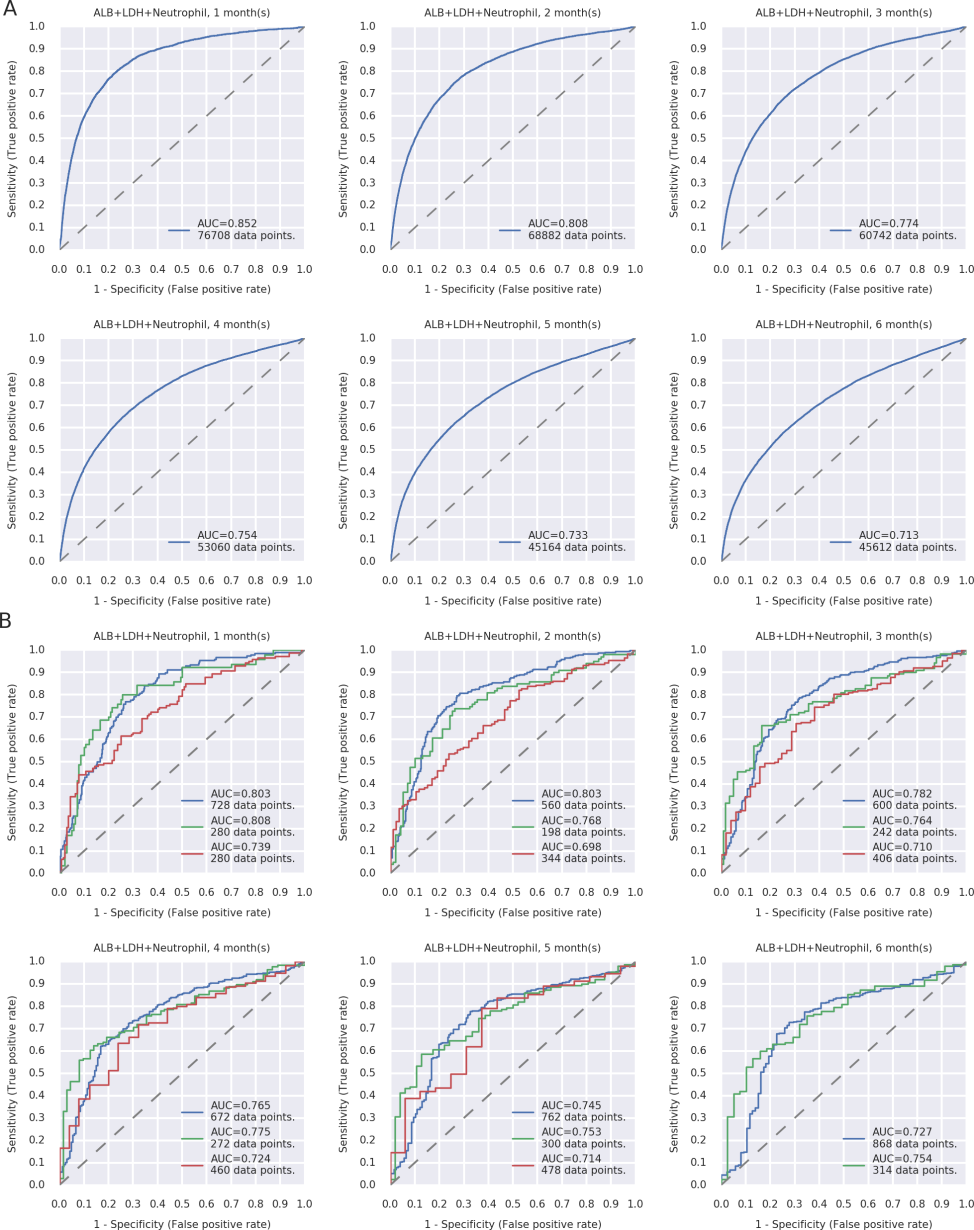
Comparison of area under the receiver operating characteristic curve (AUC) values for the prediction of death events among the (**A**) test cohort at the Kyoto University Hospital and (**B**) validation cohort (blue: Kyoto Mitsubishi Hospital, green: Kyoto Min-iren Chuo Hospital, red: J-ProVal study).

External validation was performed using data from two independent community hospitals and the J-ProVal study cohort. The outcomes from two independent community hospitals and the J-ProVal study cohort further supported the good performance of these models as AUC values of > 0.730 and > 0.700 were achieved, respectively (Fig 4) (S4 Table).

## Discussion

In this study, using time-series real-world big data, we successfully developed a set of six adaptable prognosis prediction models for patients with cancer receiving chemotherapy. Our current models allow physicians to select the optimal model according to the clinical condition of the patient and apply them repeatedly at any time point. In other words, physicians can re-estimate the prognosis of patients at any time point after the initiation of chemotherapy. We expect that our adaptable models will provide valuable information to both physicians and patients during decision-making for optimal treatment (e.g., indication of palliative chemotherapy or discontinuation of aggressive interventions or referral timing for palliative care service), which is necessary in daily clinical practice.

Previous prognosis prediction models were developed using the variables from a single time point (e.g., admission date or baseline assessment date). In contrast, our current models are based on more robust data than previous models, utilizing every laboratory variables monitored within 1 year before death events. (43 different time points per patient, Fig 1B) To the best of our knowledge, no previous studies have applied a similar method to the construction of prognosis prediction models. We have named this method as “time-series real-world big data analysis.”

With regard to statistical analysis, we selected logistic regression rather than Cox regression, which is commonly used to develop the prognostic models. Cox regression models are commonly used to estimate the survival, while logistic regression models are to predict a binary outcome. Because our current prognostic models are only for discriminating the binary survival outcomes within a specific time frame, we considered logistic regression is more feasible. In addition, we compared the prediction performance between time-dependent Cox regression models and logistic regression models in the current internal validation cohorts and found that there were no significant differences in prediction performance between logistic regression models and time-dependent Cox regression models. (S5 Table)

Our models have another unique feature. Unlike previously published models, which were designed to predict survival durations from specific time points, our models were designed to predict death events within 1–6 months and used different sets of coefficients according to the intended prediction period. For example, if we intend to predict death events within the following 6 months, we would use the model with a coefficient set at 6 months: (*p* = 1/(1 + exp(0.334 *×*_Alb_ − 0.001 *×*_LDH_ − 0.012 *×*_neutrophil_ + 0.031)) (Table 3) Similarly, predictions of death events within 3 months would require a corresponding change in the coefficient set: (*p* = 1/(1 + exp(0.482 × _Alb_ − 0.001 *×*_LDH_ − 0.017 × _neutrophil_ + 0.039)) (Table 3)

In this study, a combination of Alb, LDH, and Neu was selected from 40 explanatory variables according to the prediction performance estimated by AUC. A decrease in Alb levels reflects poor nutrition and/or hypercatabolism in patients with cancer, [14–16] whereas elevated LDH levels are closely associated with an increased tumor volume [14, 16, 17]. An elevated neutrophil count or neutrophil-to-lymphocyte ratio has also been reported as an independent prognostic factor in many studies [18, 19]. Therefore, the use of these three factors in our prediction models represents a rational choice.

Moreover, we tested the 2-item and 4-item prognostic models to compare their performance. Models using more items show better performance; however, the difference in AUC between the 3- and 4-item prognostic models was smaller than that between 2- and 3-item prognostic models. (S6 Table) Based on these results, we selected 3-item prognostic models in this study.

Our current prediction models exhibited similar levels of predictive performance across different tumor types according to AUC estimations, suggesting that the models are generalizable to all patients with cancer receiving chemotherapy. The ability to reproduce a high AUC in three independent external validation cohorts further provides strong support for the generalizability of these models.

Undoubtedly, accurate prognostic predictions facilitate the provision of optimal treatments to patients; however, physicians must handle such information carefully because some patients might not want to know about their prognoses. In this regard, the results obtained from any prognosis prediction models should be used properly in daily clinical practice in accordance with individual patients’ requests.

A potential limitation of our study is the lack of robust evidence that shows the clinical utility of prognosis prediction models including our current models. To clarify these clinical questions, we plan to assess the clinical utility of our models in a large prospective study.

In summary, by applying “time-series real-world big data analysis,” we have developed robust and adaptable prognosis prediction models for patients with cancer receiving chemotherapy. We expect that this “time-series real-world big data analysis” will be a promising tool for the future construction of prediction models for other purposes.

## Supporting information

S1 Table Patient characteristics of the validation cohorts at the Kyoto Mitsubishi, Kyoto Min-iren Chuo hospitals and the J-ProVal study. J-ProVal study, Japan prognostic assessment tools validation study.
(XLSX)

S2 Table Top 10 prediction models for the occurrence of death events within 1–3 months. ALB, albumin; ALP, alkaline phosphatise; Ca, calcium; Cl, chloride; CRP, C-reactive protein; K, potassium; LDH, lactate dehydrogenase; MCH, mean corpuscular hemoglobin; MCHC, mean corpuscular hemoglobin concentration; MCV, mean corpuscular volume; Na, sodium; PPV, positive predictive value; NPV, negative predictive value.
(XLSX)

S3 Table Top 10 prediction models for the occurrence of death events within 4–6 months. ALB, albumin; ALP, alkaline phosphatise; Ca, calcium; Cl, chloride; CRP, C-reactive protein; K, potassium; LDH, lactate dehydrogenase; MCH, mean corpuscular hemoglobin; MCHC, mean corpuscular hemoglobin concentration; MCV, mean corpuscular volume; Na, sodium; PPV, positive predictive value; NPV, negative predictive value.
(XLSX)

S4 Table External validation of albumin, lactate dehydrogenase, and neutrophil models for prediction of death events within 1–6 months. PPV, positive predictive value; NPV, negative predictive value.
(XLSX)

S5 Table Comparison of the prediction performance between time-dependent Cox regression models and logistic regression models. AUC, area under the curve; LR, logistic regression.
(XLSX)

S6 Table Comparison of prediction performance among 2-item, 3-item and 4-item prognostic models in the current internal and external validation cohorts. To save time, we used representative prognostic items to develop the 4-item prognostic models and selected albumin (Alb), neutrophil (Neu), lactate dehydrogenase (LDH), and blood urine nitrogen (BUN) prognostic models on the basis of area under the receiver operating characteristic curve (AUC) values.
(XLSX)
